# Prediction of Physical Activity Intensity with Accelerometry in Young Children

**DOI:** 10.3390/ijerph16060931

**Published:** 2019-03-15

**Authors:** Chiaki Tanaka, Yuki Hikihara, Takafumi Ando, Yoshitake Oshima, Chiyoko Usui, Yuji Ohgi, Koichi Kaneda, Shigeho Tanaka

**Affiliations:** 1 Division of Integrated Sciences, J. F. Oberlin University, Tokyo 194-0294, Japan; 2 Faculty of Creative Engineering, Chiba Institute of Technology, Chiba 275-0023, Japan; hikihara.yuki@it-chiba.ac.jp; 3 Japan Society for the Promotion of Science, Tokyo 102-0083, Japan; takafumi_andy@yahoo.co.jp; 4 Faculty of Humanities and Social Sciences, University of Marketing and Distribution Sciences, Hyogo 651-2188, Japan; Yoshitake_Oshima@red.umds.ac.jp; 5 Waseda Institute for Sport Sciences, Waseda University, Saitama 359-1192, Japan; chiyoko.koba@gmail.com; 6 Graduate School of Media and Governance, Keio University, Kanagawa 252-0882, Japan; ohgi@sfc.keio.ac.jp; 7 Faculty of Advanced Engineering, Chiba Institute of Technology, Chiba 275-0023, Japan; koichi.kaneda@p.chibakoudai.jp; 8 Department of Nutrition and Metabolism, National Institute of Health and Nutrition, National Institutes of Biomedical Innovation, Health and Nutrition, Tokyo 162-8636, Japan; tanakas@nibiohn.go.jp

**Keywords:** triaxial accelerometer, algorithm, young children, non-ambulatory activities, ambulatory activities

## Abstract

*Background*: An algorithm for the classification of ambulatory and non-ambulatory activities using the ratio of unfiltered to filtered synthetic acceleration measured with a triaxial accelerometer and predictive models for physical activity intensity (METs) in adults and in elementary school children has been developed. The purpose of the present study was to derive predictive equations for METs with a similar algorithm in young children. *Methods*: Thirty-seven healthy Japanese children (four- to six-years old) participated in this study. The five non-ambulatory activities including low-intensity activities, and five ambulatory activities were selected. The raw accelerations using a triaxial accelerometer and energy expenditure by indirect calorimetry using the Douglas bag method during each activity were collected. *Results*: For non-ambulatory activities, especially light-intensity non-ambulatory activities, linear regression equations with a predetermined intercept (0.9) or quadratic equations were a better fit than the linear regression. The equations were different from those for adults and elementary school children. On the other hand, the ratios of unfiltered to filtered synthetic acceleration in non-ambulatory activities were different from those in ambulatory activities, as in adults and elementary school children. *Conclusions*: Our calibration model for young children could accurately predict intensity of physical activity including low-intensity non-ambulatory activities.

## 1. Introduction

According to the World Health Organization guidelines on physical activity (PA), at least 60 min of moderate-to-vigorous physical activity (MVPA) every day is recommended for the health of children and adolescents [1]. Furthermore, previous systematic reviews showed that long periods of sedentary behavior (SB) were associated with adverse health outcomes in children and adolescents [2,3,4,5]. Based on such evidences, guidelines for SB in children and adolescents have been published separately from PA guidelines in several countries [6,7,8]. A systematic review concluded SB from childhood and adolescence may form the foundation for such behaviors in the future, and some types of SB may track slightly better than PA [9]. Therefore, an accurate evaluation of not only MVPA but also SB in young children is important.

Accelerometers can provide a reliable and valid estimate of energy expenditure from PA in free-living conditions [10]. According to a quantitative meta-analysis [10], many studies reported accelerometry-derived daily PA levels of preschool-age children, with cutoff values or single prediction equation for PA intensity or energy expenditure. However, the relationship between PA intensity and acceleration that accounts for ambulatory activities (e.g., walking and running) is different from that for other types of PA (e.g., playing games, playing with blocks, tossing a ball, and tidying up), as indicated in children [11,12,13,14,15,16]. Non-ambulatory activity time measured by a triaxial accelerometry contributes more to PA than ambulatory activity time in free-living preschool children [17]. Reilly et al. [18] indicated that simple approaches using the ActiGraph are not appropriate to estimate total energy expenditure in preschool-aged children against the doubly labeled water method, which may be attributed to a significant contribution of non-ambulatory activities to total energy expenditure.

Several studies to validate the estimation of PA intensity have been conducted in young children [19,20,21,22,23,24]. Predictive equations based on ambulatory activities tends to underestimate intensity of PA during non-ambulatory activities, such as playing with blocks, tossing a ball, dancing, and cleaning [11,12,13,14,15]. A specific calibration model that discriminates between non-ambulatory and ambulatory activities for young children seems useful to evaluate the SB to vigorous PAs of both non-ambulatory and ambulatory activities [14,15]. Crouter et al. [25] proposed a two-regression model for children aged 8 to 15 years to discriminate between ambulatory activities and non-ambulatory activities, based on the variability of the acceleration. In our previous study, we suggested a new calibration model discriminating ambulatory movements from non-ambulatory activities with a triaxial accelerometer for adults and elementary school children using the ratio of raw synthetic acceleration to filtered synthetic acceleration without gravity acceleration from light to vigorous activities [16,26]. However, the calibration models for adults or elementary school children are expected not to be suitable for estimating the PA of young children, because growth and maturation level, muscle activity, and the poorer economy of movement compared to adults or elementary school children affect the relationship between acceleration and PA intensity [22]. Besides, because children’s habitual PA behavior is likely to be more complex, additional activities such as playing with blocks and ball throwing have sometimes been included in calibrating tasks [16]. To our knowledge, however, no studies have proposed a calibration model for young children with an algorithm classifying non-ambulatory activities and ambulatory movements except our study using ActivTracer, a triaxial accelerometer. For adults, the algorithm using the ratio of raw synthetic acceleration to filtered synthetic acceleration without gravity acceleration appears to be better than the other algorithm used with ActivTracer [26].

The aims of the present study were to propose a new calibration model with an algorithm classifying ambulatory and non-ambulatory activity to evaluate young children’s PA, according to the procedure of our previous study for adults and elementary school children, and to compare the calibration model and algorithm with those for young children and adults or elementary school children.

## 2. Materials and Methods

Thirty-seven healthy young Japanese children (19 boys and 18 girls) attending kindergarten were invited to participate in this study via public advertisements. The physical characteristics of the participants are shown in Table 1. None of the participants had any physical impairments that could affect daily life activity or energy metabolism. The purpose of the study was explained to all participants and children’s parents, and all participants’ parents provided written informed consent prior to the beginning of the study according to the guidelines of the Declaration of Helsinki. All procedures involving human subjects were approved by the Ethical Committee of J. F. Oberlin University (No. 09005).

### 2.1. Anthropometry

Body weight was measured to the nearest 0.1 kg with a digital balance, and body height on a stadiometer to the nearest 0.1 cm. Body mass index (kg/m^2^) was calculated as body weight in kilograms divided by body height in meters squared.

### 2.2. Procedures

To avoid the effect of diet-induced thermogenesis, participants visited the laboratory two hours after breakfast. Anthropometric measurements were taken after explaining the study protocol. Next, the participants rested for 30 min, and then resting metabolic rate (RMR) was measured with the participant in the sitting position for 14 min and while watching a children’s video to avoid fidgeting [27]. After the measurement of RMR, participants performed 9 different physical activities for approximately 3 to 7 min, with energy expenditure being measured throughout. All participants wore a triaxial accelerometer on the waist, tightly attached with a belt, during each activity. The accelerometers were synchronized with a wave clock before the start of the experiment.

### 2.3. Triaxial Accelerometer

A triaxial accelerometer device (74 mm × 46 mm × 34 mm and weighing 60 g including batteries) with 4 GB of memory (Omron Healthcare, Kyoto, Japan) consisting of a micro electro mechanical system-based accelerometer (LIS3LV02DQ; ST-Microelectronics, Geneva, Switzerland), which responds to, not only acceleration due to movement but also gravitational acceleration, was used. This device could memorize the synthetic acceleration using a measurement range of ±6 G and a resolution of 3 mG. Accelerations in the vertical (*x*), anteroposterior (*y*), and mediolateral (*z*) axes with each activity were detected at a rate of 32 Hz. Each of the three signals obtained from the accelerometer was passed through a high-pass filter with a cut-off of 0.7 Hz (filtered acceleration), in order to exclude gravitational acceleration component. The integral of the absolute value of the acceleration was calculated, and then the synthetic acceleration (vector magnitude: (X2 + Y2 + Z2)^0.5^ was obtained. This accelerometer is a prototype of commercial products (Active style Pro HJA-350IT and HJA-750C; Omron Healthcare, Kyoto, Japan).

### 2.4. Indirect Calorimetry

Expired gas was collected using the Douglas bag method. Each participant was fitted with a facemask and breathed into a Douglas bag. The gas concentrations of oxygen and carbon dioxide were analyzed by a mass spectrometer (ARCO-2000; Arco System Inc., Kashiwa, Japan), and expired gas volume using a certified dry gas meter (DC-5; Shinagawa Co., Ltd., Tokyo, Japan). From oxygen consumption and carbon dioxide production, the energy expenditure (EE) of each activity was calculated using Weir’s equation [28]. In addition, to calculate the metabolic equivalent (MET) values, the EE during each activity was divided by the measured value for the RMR.

### 2.5. Selection of Physical Activity and Sedentary Behaviour for Calibration Models

The selected activities were watching a video while seated for 14 min (RMR), coloring for 6 min, playing in a sand box for 5 min, tidying up for 3 min, tossing a ball for 4 min, normal (55 m/min) and brisk walking (70 m/min) according to a pace leader for 4 min each, stair climbing (down and up) for 4 min between the third basement floor and the fourth floor and jogging (110 m/min) for 3 min. These activities were chosen as representative activities of daily life, based on our observations in a preliminary study using the activity records of observers of 4- to 6-year-old children in a nursery school. Moreover, the selected activities in the present study were able to be conducted with a facemask and a Douglas bag attached to 4- to 6-year-old children. A preliminary period was prepared for participants to reach a steady-state condition with 2 min in the beginning of the measurement of each activity. Participants performed the sequence of tasks with a recovery period between tasks.

### 2.6. Discrimination Method

As in our previous study which reported an algorithm for the classification of non-ambulatory (household) and ambulatory activities by the ratio (e.g., cut-off value for adults, 1.16) of unfiltered synthetic acceleration to filtered synthetic acceleration [29], gravitational acceleration was removed from the filtered synthetic acceleration [16,26,29]. The sensitivity of discrimination was 98.7% or 99.1% for each of our 11 selected activities for adults and elementary school children, respectively. In addition, the ratio (1.16) is used for the commercially available accelerometers (Activity style Pro HJA-350IT (Omron Healthcare, Kyoto, Japan) and HJA-750C (Omron Healthcare, Kyoto, Japan)) and a lot of papers using these accelerometers have been published from Japan (e.g., [30,31,32,33,34,35,36]). Therefore, this discriminative procedure was applied to the young children’s calibration model in the present study.

### 2.7. Analyses

Statistical analysis was performed with SPSS version 23.0 J for Windows (SPSS Inc, Tokyo, Japan). All results are shown as mean ± standard deviation (SD). The multiple regression models with a dependent variable of MET value for ambulatory or non-ambulatory activities to examine the effects of sex and age on measured MET values were used. The determination coefficient (*R*^2^) and the estimated standard error (SEE) was used to evaluate the relationships between MET values as a dependent variable and filtered synthetic acceleration and sex or age as independent variables. In addition, the multiple regression analyses with a predetermined intercept (0.9) and a dependent variable of MET value, hypothesizing that a MET value without movement during awakening is 0.9, were performed. This is because the intercept of the regression line between MET values and accelerations, and the ratio of energy expenditure between sitting quietly and lying quietly in adults [26,37] and in children [38] have been reported to be around 0.9. Stair climbing down and up are ambulatory activities, but these were excluded when the regression models were developed, because the MET values of these activities were far from the regression lines of the ambulatory activities. A quadratic model with filtered synthetic acceleration and the square was also applied for non-ambulatory activities. We examined the validity of the algorithms for adults [26] or elementary school children [16] established in previous papers. Mean differences and limits of agreement between predicted and measured MET values are indicated by Bland and Altman plots [39]. *p* < 0.05 was considered statistically significant.

## 3. Results

### 3.1. Characteristics of the Study Participants and Observed Energy Expenditure, Metabolic Equivalents (MET), and Accelerations for Each Activity

**Figure 1:**
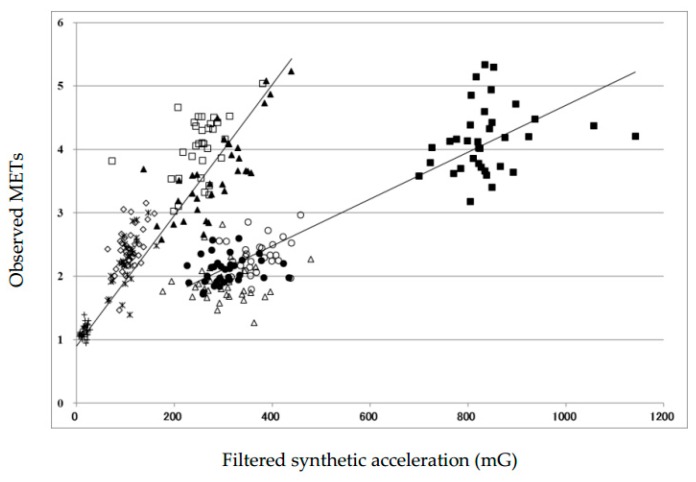
Correlation diagram of filtered synthetic accelerometer counts and MET during ambulatory and non-ambulatory activities. Simple regression lines are indicated (see Table 3).

The characteristics of the participants are shown in Table 1. Discrimination with the ratio of unfiltered to filtered synthetic accelerations provided a highly correct discrimination when the value of the ratio was 1.16 were calculated, and the cutoff value was obtained for adults (Table 2). Therefore, we estimated METs through simple equations according to the results of discrimination with the ratio of 1.16, and then compared these values with the measured METs. The relationship of synthetic acceleration to MET values in Figure 1 was described. It indicates that the plots for non-ambulatory activities are different from those for ambulatory movements. In addition, the plots for stair-climbing down and up were far apart from the regression lines. A strong linear relationship in both equations, except for stair-climbing down and up, could be confirmed.

### 3.2. Regression Equations of Estimating Metabolic Equivalent in Non-Locomotive and Locomotive Activities Except Stair Climbing

Based on a simple multiple regression model for non-ambulatory activities (Equation (1)), the MET value for coloring was overestimated by 0.25 ± 0.10 (23.0 ± 10.3%) (Table 3). Therefore, a multiple regression model with a fixed intercept (0.9) was applied (Equation (2)). As a result, the prediction error of coloring was much improved to −0.01 ± 0.12 (0.5 ± 10.9%), with comparable prediction errors for other non-ambulatory activities. When sex or age was added as an independent variable, the prediction accuracy improved slightly (Equations (3) and (4)). However, a quadratic equation with an intercept of 0.9 and without sex or age as an independent variable (Equation (5)) was a better fit than the linear regressions. A multiple regression model with sex or age had little improvement for the prediction of ambulatory activities.

### 3.3. Percent Differences between the Predicted and the Observed MET Values

**Figure 2:**
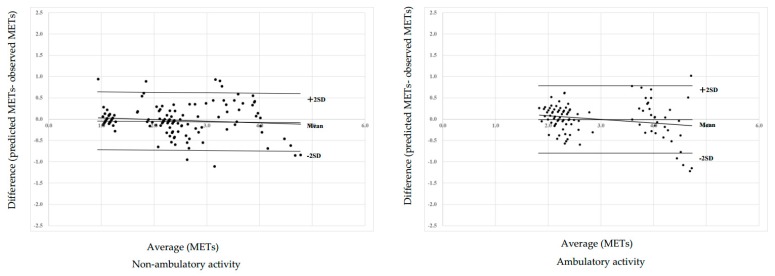
Differences between the predicted and measured METs from each equation by Bland and Altman plot analysis.

In order to examine the validity of the algorithms for adults or elementary school children, the estimated MET values using equations for adults or elementary school children were compared with measured MET values during each activity (Table 4). As a result, the predicted values of tidying up (−0.18 ± 0.32, −7.1 ± 11.0%) and ascending stairs (−2.10 ± 0.44, −51.6 ± 6.0%) were significantly underestimated against the measured values. In contrast, the predicted value of descending stairs (0.23 ± 0.37, 14.8 ± 21.7%) was significantly overestimated. The equations for elementary school children without adjustment for age or equations for adults significantly overestimated the MET value, and the equation for elementary school children with adjustment for age significantly underestimated the MET value, except for ascending stairs (Table 4). In addition, the differences between the measured MET values and the predicted MET values from Equation (5) (quadratic equation) or Equation (6) by Bland and Altman plots were described (Figure 2). As a result, non-ambulatory activity showed a mean difference of −0.03 MET values and limits of agreement (±2 SD) from +0.66 to −0.72 MET values. The ambulatory activity equation showed a mean difference of +0.00 MET values and limits of agreement (±2 SD) from +0.79 to −0.78 MET values.

## 4. Discussion

In this study, the predictive equations to predict METs from synthetic acceleration (mG) in both non-ambulatory and ambulatory activities, except for climbing down and up stairs, in young children were developed. For non-ambulatory activity, a quadratic equation was a better fit than the linear regression, especially for light-intensity non-ambulatory activities. The equations for young children obtained in the present study were different from those previously published for elementary school children [16] and adults [26]. The ratios of unfiltered to filtered synthetic acceleration in non-ambulatory activities were different from those in ambulatory activities, and the same cutoff value as that for adults could be applied for young children. Using the algorithm proposed in the present study, PA for young children, including SB, can be estimated much more accurately than those for adults or elementary school children. As a result, PA status of young children can be accurately monitored and the determinants of PA or effects of PA interventions can be examined, with the accelerometer and proposed algorithms in the present study.

The first finding suggests that the discrimination procedures developed for adults, and even for elementary school children, is applicable to young children with various activity components and patterns. In our previous studies, we found that the percentages of correct discrimination with the algorithm in elementary school children and adults was remarkable, at 99.1% and 98.7%, respectively, when the ratio of unfiltered to filtered synthetic acceleration was 1.12 and 1.16, respectively [16,26]. In the present study, the rate of correct discrimination using the threshold of discrimination for adults (1.16) was also excellent for young children (Table 2). The rate of the ratio over 1.16 was 82.4% for tidying up, but this may be due to the complex movement of the activity. Compared with the discrimination method that used the coefficient of variation in a previous study (97% for ambulatory activities and 89.5% for non-ambulatory activities) [25], the rate of correct discrimination by our discrimination procedure seems better. The mean difference in estimation accuracy of the PA intensity by our calibration model for young children was −0.02 METs and limits of agreement (0.36 SD) from +0.71 to −0.75 METs, except for stairs, were similar to the results we obtained in our previous studies for elementary school children and adults [16,26]. These results suggest that our specific models were well suited to evaluate the PA even for young children. There was a large underestimation of METs for ascending stairs; however, the time spent on this activity is likely short, so the obtained prediction error would result in a small prediction error for total energy expenditure.

One feature of the present study is that multiple regression models with a fixed intercept (0.9) were applied, which contributed to a much lower prediction error for coloring (−0.01 ± 0.12 (0.5 ± 10.9%)) using Equation (5). Low-intensity activity, such as SB, occupies a large percentage of daily life, so even a small prediction error of 0.25 ± 0.10 METs (23.0 ± 10.3%), produced by a simple regression model (Equation (1)), may cause a large error for the estimation of total energy expenditure. In addition, such an error in low-intensity activities may influence the prediction of sedentary time or the number of breaks in SB. Moreover, the quadratic equation with an intercept of 0.9 (Equation (5)) enabled prediction of non-ambulatory PA with higher intensity.

In the present study, we found that the adjusted *R*^2^ and the SEE were slightly better when sex was added as an independent variable into the standard predictive equation for non-ambulatory or ambulatory activity (Table 3). On the other hand, *R*^2^ and SEE in the predictive equation with quadratic filtered synthetic acceleration (Equation (5)) were the best in equations for non-ambulatory activity (Table 3). This would mean that it might not be necessary to control for weight, age, and sex, similar to several other calibration studies, and the relationship is non-linear [10,14,40]. Moreover, the equations were different from those for elementary school children and adults. Estimation of METs using equations for young children appears better than METs using equations indicated in previous reports in elementary school children and adults (Table 4). One of the reasons may be that the PA behaviors in children are complicated and less economical [41] and are not continuous [42]. On the other hand, it should be considered that each calibration model is based on slightly different physical activities between the present study and previous studies in elementary school children and adults. There were several limitations to the current study. It is necessary to examine the accuracy of different PAs from those tested in the present study, because selected PAs were common in daily life for young children, but these PAs do not cover the daily activities of young children. The models proposed in the present study should be cross-validated in different groups of young children to examine the robustness. Furthermore, the developed models for estimating PA intensity must be validated under conditions close to free-living in the future.

## 5. Conclusions

The specific calibration model that discriminates between non-ambulatory and ambulatory activities and uses a quadratic equation for non-ambulatory activities for young children can be useful for various types of PAs, including SB and light-intensity non-ambulatory activities.

## Figures and Tables

**Table 1:** Physical characteristics of subjects.

Variable	Mean ± SD
Age (yr)	6.1 ± 0.6
Height (cm)	113.4 ± 5.6
Weight (kg)	20.5 ± 2.9
BMI (kg/m^2^)	15.9 ± 1.5

BMI: body mass index, SD: standard deviation.

**Table 2:** Observed energy expenditure, metabolic equivalents (MET), and accelerations for each activity.

Activity	*n*	Energy Expenditure	MET	Filtered Acceleration	Ratio of Unfiltered to Filtered Synthetic Accelerations	Ratio (Filtered to Unfiltered Accelerations)	
		(kJ min^−1^)		Synthetic (mG)		>1.16 (%)	<1.16 (%)
Watching a video while seated	37	3.06 ± 0.42	1.14 ± 0.10	7.1 ± 4.3	2.55 ± 0.61	100.0	0.0
Coloring	33	3.51 ± 0.51		16.3 ± 5.9	2.57 ± 0.45	100.0	0.0
Playing in a sand box	33	6.82 ± 1.39	2.23 ± 0.38	102.3 ± 20.9	2.10 ± 0.33	100.0	0.0
Tidying up	33	7.58 ± 1.39	2.46 ± 0.35	109.4 ± 21.2	2.06 ± 0.48	82.4	17.6
Tossing a ball	34	11.10 ± 2.83	3.63 ± 0.68	283.7 ± 70.0	1.29 ± 0.13	100.0	0.0
Normal walking	34	6.39 ± 0.97	2.10 ± 0.22	306.1 ± 48.5	1.00 ± 0.01	0.0	100.0
Brisk walking	31	7.27 ± 0.85	2.36 ± 0.27	373.1 ± 47.5	1.00 ± 0.01	0.0	100.0
Jogging	35	12.66 ± 1.94	4.16 ± 0.53	838.9 ± 83.3	1.02 ± 0.01	0.0	100.0
Ascending stairs	32	6.17 ± 1.31	4.03 ± 0.49	251.7 ± 49.3	1.12 ± 0.24	0.0	100.0
Descending stairs	34	12.46 ± 2.42	2.00 ± 0.31	301.4 ± 58.6	1.05 ± 0.02	6.3	93.7

Mean ± standard deviation, METs: metabolic equivalents. METs were calculated as energy expenditure for each activity divided by energy expenditure for resting in the sitting position.

**Table 3:** Regression equations of estimating metabolic equivalent in non-locomotive and locomotive activities except stair climbing.

Type of Equation	Regression Equation	SEE	*R* ^2^	*p*
Non-ambulatory activity				
(1) Simple regression without a predetermined intercept	METs = 1.2459 + 0.0087 × filtered synthetic accelerometer counts	0.370	0.860	*
Regression with a predetermined intercept (0.9)				
(2) Filtered synthetic acceleration	METs = 0.0103 × filtered synthetic accelerometer counts + 0.9	0.428	0.942	*
(3) Filtered synthetic acceleration with sex	METs = 0.0089 × filtered synthetic accelerometer counts + 0.2180 × sex + 0.9	0.361	0.959	*
(4) Filtered synthetic acceleration with age	METs = 0.0087 × filtered synthetic accelerometer counts + 0.0567 × age + 0.9	0.370	0.956	*
(5) Quadratic filtered synthetic acceleration	METs = 0.0144 × filtered synthetic accelerometer counts − 0.0000147 × (filtered synthetic accelerometer counts)^2^ + 0.9	0.350	0.961	*
Ambulatory activity				
(6) Filtered synthetic acceleration	METs = 1.0012 + 0.00370 × filtered synthetic accelerometer counts	0.391	0.847	*
(7) Filtered synthetic acceleration with sex	METs = 0.7585 + 0.00368 × filtered synthetic accelerometer counts + 0.1706 × sex	0.383	0.853	*
(8) Filtered synthetic acceleration with age	METs = 0.8310 + 0.00370 × filtered synthetic accelerometer counts + 0.0278 × age	0.393	0.846	*

METs: metabolic equivalents, SEE: standard error of estimate, *: *p* < 0.05.

**Table 4:** Percent differences between the predicted and the observed metabolic equivalents.

Equation	Present Study *^1^		Present Study*^2^		For elementary School Children *^3^		For adults *^4^	
Activity	Absolute Difference	% Difference	Absolute Difference	% Difference	Absolute Difference	% Difference	Absolute Difference	% Difference
Coloring	0.25 ± 0.10	23.0 ± 10.3	−0.01 ± 0.12	0.5 ± 10.9	0.30 ± 0.11	26.8 ± 11.2	0.32 ± 0.20	28.5 ± 19.1
Playing in a sand box	−0.01 ± 0.10	0.1 ± 8.4	−0.02 ± 0.29	0.8 ± 16.1	0.32 ± 0.29	16.6 ± 18.4	1.12 ± 0.34	52.9 ± 24.3
Tidying up	0.08 ± 0.30	5.9 ± 17.1	−0.18 ± 0.32	−7.1 ± 11.0	0.18 ± 0.33	9.0 ± 15.6	1.03 ± 0.40	43.7 ± 21.0
Tossing a ball	0.00 ± 0.44	1.6 ± 12.6	0.08 ± 0.50	3.9 ± 14.2	1.28 ± 0.59	36.5 ± 18.3	2.64 ± 1.41	73.2 ± 38.3
Normal walking	−0.10 ± 0.31	2.5 ± 13.9	−0.10 ± 0.31	−2.5 ± 13.9	0.37 ± 0.30	18.6 ± 14.7	1.64 ± 0.44	79.5 ± 23.8
Brisk walking	0.03 ± 0.27	2.2 ± 12.0	0.03 ± 0.27	2.2 ± 12.0	0.45 ± 0.30	20.5 ± 14.6	1.97 ± 0.41	85.3 ± 23.3
Jogging	−0.05 ± 0.55	0.1 ± 12.7	−0.05 ± 0.55	0.1 ± 12.7	0.98 ± 0.60	25.3 ± 16.2	4.17 ± 0.80	103.0 ± 26.7
Ascending stairs	−2.10 ± 0.44	−51.6 ± 6.03	−2.10 ± 0.44	−51.6 ± 6.0	−1.83 ± 0.44	−44.9 ± 7.2	0.62 ± 0.83	−14.2 ± 22.9
Descending stairs	0.23 ± 0.37	14.8 ± 21.7	0.23 ± 0.37	14.8 ± 21.7	0.56 ± 0.42	33.0 ± 26.6	−1.82 ± 0.59	101.1 ± 42.4

*^1^ The simple regression model without a preset intercept was used in non-ambulatory activity and only the filtered synthetic acceleration was used in ambulatory activity. *^2^ The quadratic filtered synthetic acceleration was used in non-ambulatory activity and only the filtered synthetic acceleration was used in ambulatory activity (same as ^*1^ in ambulatory activity). *^3^ Hikihara et al. [16], *^4^ Ohkawara et al. [26]. Mean ± standard deviation.

## References

[1] Global Recommendations on Physical Activity for Health. http://www.who.int/dietphysicalactivity/publications/physical-activity-recommendations-5-17years.pdf?ua=1.

[2] Pate R.R., Mitchell J.A., Byun W., Dowda M. (2011). Sedentary behaviour in youth. Br. J. Sports Med..

[3] Salmon J., Tremblay M.S., Marshall S.J., Hume C. (2011). Health risks, correlates, and interventions to reduce sedentary behavior in young people. Am. J. Prev. Med..

[4] LeBlanc A.G., Spence J.C., Carson V., Connor Gorber S., Dillman C., Janssen I., Kho M.E., Stearns J.A., Timmons B.W., Tremblay M.S. (2012). Systematic review of sedentary behaviour and health indicators in the early years (aged 0–4 years). Appl. Physiol. Nutr. Metab..

[5] Tanaka C., Reilly J.J., Huang W.Y. (2014). Longitudinal changes in objectively measured sedentary behaviour and their relationship with adiposity in children and adolescents: Systematic review and evidence appraisal. Obes. Rev..

[6] Start Active, Stay Active A Report on Physical Activity from the Four Home Countries’ Chief Medical Officers. https://www.sportengland.org/media/2928/dh_128210.pdf.

[7] Canadian Society for Exercise Physiology Canadian 24-Hour Movement Guidelines for Children and Youth (Aged 5–17). http://www.csep.ca/en/guidelines/get-the-guidelines.

[8] Australian Governments, Department of Health Australia’s Physical Activity and Sedentary Behaviour Guidelines. http://www.health.gov.au/internet/main/publishing.nsf/content/health-pubhlth-strategphys-act-guidelines.

[9] Biddle S.J., Pearson N., Ross G.M., Braithwaite R. (2010). Tracking of sedentary behaviours of young people: A systematic review. Prev. Med..

[10] Bornstein D.B., Beets M.W., Byun W., McIver K. (2011). Accelerometer-derived physical activity levels of preschoolers: A meta-analysis. J. Sci. Med. Sport.

[11] Puyau M.R., Adolph A.L., Vohra F.A., Zakeri I., Butte N.F. (2004). Prediction of activity energy expenditure using accelerometers in children. Med. Sci. Sports Exerc..

[12] Rowlands A.V., Thomas P.W., Eston R.G., Topping R. (2004). Validation of the RT3 triaxial accelerometer for the assessment of physical activity. Med. Sci. Sports Exerc..

[13] Freedson P., Pober D., Janz K.F. (2005). Calibration of accelerometer output for children. Med. Sci. Sports Exerc..

[14] Tanaka C., Tanaka S., Kawahara J., Midorikawa T. (2007). Triaxial accelerometry for assessment of physical activity in young children. Obesity (Silver Spring).

[15] Kawahara J., Tanaka S., Tanaka C., Aoki Y., Yonemoto J. (2011). Estimation of daily inhalation rate in preschool children using a tri-axial accelerometer: A pilot study. Sci. Total Environ..

[16] Hikihara Y., Tanaka C., Oshima Y., Ohkawara K., Ishikawa-Takata K., Tanaka S. (2014). Prediction models discriminating between nonlocomotive and locomotive activities in children using a triaxial accelerometer with a gravity-removal physical activity classification algorithm. PLoS ONE.

[17] Tanaka C., Tanaka S. (2009). Daily physical activity in Japanese preschool children evaluated by triaxial accelerometry: The relationship between period of engagement in moderate-to-vigorous physical activity and daily step counts. J. Physiol. Anthropol..

[18] Reilly J.J., Kelly L.A., Montgomery C., Jackson D.M., Slater C., Grant S., Paton J.Y. (2006). Validation of Actigraph accelerometer estimates of total energy expenditure in young children. Int. J. Pediatr. Obes..

[19] Reilly J.J., Coyle J., Kelly L., Burke G., Grant S., Paton J.Y. (2003). An objective method for measurement of sedentary behavior in 3- to 4-year olds. Obes Res..

[20] Pfeiffer K.A., McIver K.L., Dowda M., Almeida M.J., Pate R.R. (2006). Validation and calibration of the Actical accelerometer in preschool children. Med. Sci. Sports Exerc..

[21] Adolph A.L., Puyau M.R., Vohra F.A., Nicklas T.A., Zakeri I.F., Butte N.F. (2012). Validation of uniaxial and triaxial accelerometers for the assessment of physical activity in preschool children. J. Phys. Act. Health.

[22] Butte N.F., Wong W.W., Lee J.S., Adolph A.L., Puyau M.R., Zakeri I.F. (2014). Prediction of energy expenditure and physical activity in preschoolers. Med. Sci. Sports Exerc..

[23] Janssen X., Cliff D.P., Reilly J.J., Hinkley T., Jones R.A., Batterham M., Ekelund U., Brage S., Okely A.D. (2014). Validation and calibration of the activPAL™ for estimating METs and physical activity in 4–6 year olds. J. Sci. Med. Sport.

[24] Johansson E., Larisch L.M., Marcus C., Hagströmer M. (2016). Calibration and validation of a wrist- and hip-worn Actigraph accelerometer in 4-year-old children. PLoS ONE.

[25] Crouter S.E., Horton M., Bassett D.R. (2012). Use of a two-regression model for estimating energy expenditure in children. Med. Sci. Sports Exerc..

[26] Ohkawara K., Oshima Y., Hikihara Y., Ishikawa-Takata K., Tabata I., Tanaka S. (2011). Real-time estimation of daily physical activity intensity by a triaxial accelerometer and a gravity-removal classification algorithm. Br. J. Nutr..

[27] Amorim P.R., Byrne N.M., Hills A.P. (2007). Combined effect of body position, apparatus and distraction on children’s resting metabolic rate. Int. J. Pediatr. Obes..

[28] Weir J.B. (1949). New methods for calculating metabolic rate with special reference to protein metabolism. J. Physiol..

[29] Oshima Y., Kawaguchi K., Tanaka S., Ohkawara K., Hikihara Y., Ishikawa-Takata K., Tabata I. (2010). Classifying household and locomotive activities using a triaxial accelerometer. Gait Posture.

[30] Tanaka C., Reilly J.J., Tanaka M., Tanaka S. (2016). Seasonal changes in objectively measured sedentary behavior and physical activity in Japanese primary school children. BMC Public Health.

[31] Tanaka C., Tanaka M., Okuda M., Inoue S., Aoyama T., Tanaka S. (2017). Association between objectively evaluated physical activity and sedentary behavior and screen time in primary school children. BMC Res. Notes.

[32] Tanaka C., Reilly J.J., Tanaka M., Tanaka S. (2018). Changes in Weight, Sedentary Behaviour and Physical Activity during the School Year and Summer Vacation. Int. J. Environ. Res. Public Health.

[33] Suzuki I., Okuda M., Tanaka M., Inoue S., Tanaka S., Tanaka C. (2018). Variability in school children’s activity occurs in the recess and before-school periods. Pediatr. Int..

[34] Tanaka C., Tanaka M., Tanaka S. (2018). Objectively evaluated physical activity and sedentary time in primary school children by gender, grade and types of physical education lessons. BMC Public Health.

[35] Aoyama T., Tanaka S., Tanaka M., Okuda M., Inoue S., Tanaka C. (2018). Association between age at onset of independent walking and objectively measured sedentary behavior is mediated by moderate-to-vigorous physical activity in primary school children. PLoS ONE.

[36] Tanaka C., Okuda M., Tanaka M., Inoue S., Tanaka S. (2018). Associations of Physical Activity and Sedentary Time in Primary School Children with Their Parental Behaviors and Supports. Int. J. Environ. Res. Public Health.

[37] Taguri E., Tanaka S., Ohkawara K., Ishikawa-Takata K., Hikihara Y., Miyake R., Yamamoto S., Tabata I. (2010). Validity of physical activity indices for adjusting energy expenditure for body size: Do the indices depend on body size?. J. Physiol. Anthropol..

[38] Ridley K., Ainsworth B.E., Olds T.S. (2008). Development of a compendium of energy expenditures for youth. Int. J. Behav. Nutr. Phys. Act..

[39] Bland J.M., Altman D.G. (1986). Statistical methods for assessing agreement between two methods of clinical measurement. Lancet.

[40] Treuth M.S., Schmitz K., Catellier D.J., McMurray R.G., Murray D.M., Almeida M.J., Going S., Norman J.E., Pate R. (2004). Defining accelerometer thresholds for activity intensities in adolescent girls. Med. Sci. Sports Exerc..

[41] Allor K.M., Pivarnik J.M., Sam L.J., Perkins C.D. (2000). Treadmill economy in girls and women matched for height and weight. J. Appl. Physiol..

[42] Bailey R.C., Olson J., Pepper S.L., Porszasz J., Barstow T.J., Cooper D.M. (1995). The level and tempo of children’s physical activities: An observational study. Med. Sci. Sports Exerc..

